# Göttingen minipigs present with significant regeneration kinetics after sphincter injury compared to German landrace gilts; a feasibility study

**DOI:** 10.1186/s12917-025-04529-x

**Published:** 2025-02-18

**Authors:** Jasmin Knoll, Niklas Harland, Bastian Amend, Arnulf Stenzl, Wilhelm K. Aicher

**Affiliations:** 1 Centre of Medical Research, Department of Urology, University of Tuebingen Hospital, Eberhard-Karls-University, 72072 Tuebingen, Germany; 2 Department of Urology, University of Tuebingen Hospital, Eberhard-Karls-University, 72076 Tuebingen, Germany; 3 Zentrum fuer Medizinische Forschung, Urologische Universitaetsklinik, Waldhoernlestr. 22, Tuebingen, D-72072 Germany

**Keywords:** Wound healing animal model, Urinary incontinence, Sphincter deficiency, Göttingen minipigs, Landrace pigs

## Abstract

**Background:**

Animals serve as important models for exploring the pathology, diagnosis, and therapy of different diseases and injuries. While smaller animals are preferred for bulk cohort studies, larger animals offer opportunities to investigate surgical procedures at proportions close to the human situation. Therefore, in a feasibility study, we investigated urethral sphincter deficiency in German landrace gilts and Göttingen minipigs to develop a model of urinary incontinence as a basis for future preclinical studies of incontinence therapies. Urethral sphincter deficiency was induced surgically by transurethral electrocautery and balloon dilatation, and the deficiency was determined by urodynamics after injury and during follow-up. In cryosections, sphincter injury was visualized by histochemistry.

**Results:**

Sphincter deficiency was induced in two cohorts and groups of four female Göttingen minipigs each (total *n* = 20) by two different treatments. One cohort of minipigs showed an initially significant urethral sphincter deficiency (treatment 1; n = 16, *p* < 0.001). However, spontaneous sphincter regeneration was observed within one to two weeks. The other cohort of minipigs (treatment 2; *n* = 4) displayed a non-significant reduction of urethral sphincter pressure and an increase in muscle strength over time as well. In contrast, German landrace gilts presented immediately after treatment with significant sphincter deficiency (treatment 1; *n* = 6, 21%, *p* < 0.001) and suffered from significant loss of sphincter function for at least five weeks (67%, *p* < 0.01).

**Conclusion:**

Göttingen minipigs inherit significantly superior sphincter regeneration capacities compared to landrace pigs. This difference may bias preclinical studies in urology and other fields and explain in part seemingly contradictory results from different animal studies.

## Background

Urinary incontinence (UI) is a significant personal, social, and medical challenge. A recent meta-analysis reported a mean UI prevalence of 41% (range 9% – 75%), and 26% of women reported a daily loss of urine [1]. In women, UI is associated with pregnancy, vaginal delivery, and hormonal changes in menopause [2, 3], while in men injuries after prostate cancer surgery predominate [4]. In some cases, physical exercise of lower pelvic floor muscles may improve the situation, sometimes complemented by electrophysiological stimulation, bio-feedback devices, or chemicals [5]. If these therapies fail to yield improvement, surgical therapies including cell therapies can be considered [6]. However, studies reporting on cell therapy of UI grant a quite diverse picture. In several preclinical animal studies, advantages of cell therapies were reported but cohort sizes were often small, follow-up times were short, or studies did not yield an overall significant outcome [7–10]. Some clinical studies suffered from similar problems [11–13], other studies were retracted (not cited) or not published nor reported to the authorities (see e.g., European Medicines Agency (EMA) Registry on cell therapy of stress urinary incontinence (SUI)). However, some recent papers rated the efficacy of UI cell therapies over other treatments [14, 15], while others reported little benefit [16]. Robust and relevant large animal models of human diseases or deficiencies investigating the efficacies and kinetics of different UI therapies are essential to solving these issues [17–23].

**Fig. 1:**
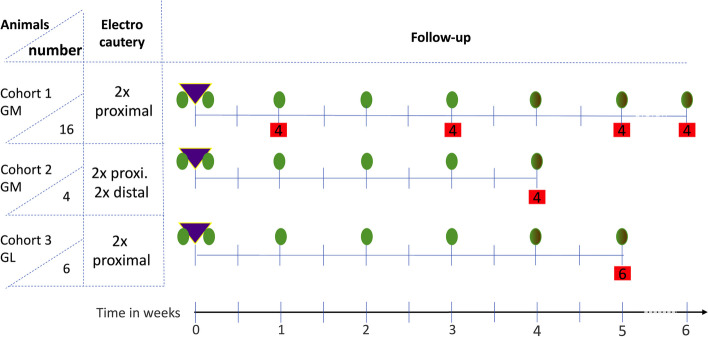
Schematic overview of animal experiments. Timelines of surgically induced urethral sphincter deficiency were investigated in three animal cohorts. Sphincter deficiency was induced in cohort 1 (sixteen GM gilts total) by two proximal electrocauteries and balloon dilatation. The overall follow-up lasted for six weeks. After 1, 3, 5, and 6 weeks 4 GM each were taken out of the study to prepare sphincter samples for histology as indicated by numbers in red boxes. Cohort 2 (four GM gilts) was treated by two proximal and two distal electrocauteries and balloon dilatation. The overall follow-up lasted for four weeks. After sacrifice, urethral sphincter tissue was analyzed by histology. Cohort 3 (six GL gilts) were treated by two proximal electrocauteries and balloon dilatation as cohort 1 animals. The overall follow-up lasted for five weeks. After sacrifice, urethral sphincter tissue was analysed by histology. Urodynamics (green ovals) were performed on day 0 immediately before and immediately after induction of sphincter deficiency (violet triangles) and weekly thereafter as indicated

A variety of UI animal models were introduced in the field, ranging from rodents [24, 25] to farm animals [8, 17], and non-human primates [26, 27]. Rodents facilitate studies with larger cohorts and grant statistical advantages for regimens with small molecules (chemicals, proteins) but come with an entirely different anatomical situation compared to human patients. For instance, precise needle injections of active components in a rodent’s urethra require excellent surgical skills. Moreover, in rodents, transurethral application of medication and transurethral follow-up seem impossible. In contrast, the anatomy and build of *Macaca fascicularis*, a non-human primate, comes closer to the human situation, and studies in cynomolgus monkeys provided results in favor of UI cell therapy [28]. However, this animal model is too small to develop working prototypes of surgical instruments intended for later clinical use. We, therefore, extended our experimental work with gilts as UI models, as they provide size and metabolism suitable for preclinical studies with standard-size surgical instruments [17, 20, 21, 23]. However, the rapid growth of young landrace pigs may cause technical difficulties for long-term follow-up of UI therapy. Hence, in a feasibility study, we investigated if a combination of transurethral injury of the sphincter complex plus urethral dilatation produced a significant and lasting sphincter deficiency in Göttingen minipigs (GM). The GM were selected based on their slow growth kinetics leveling off at a mean weight of 45 ± 15 kg at about 20 months of age, thus facilitating long-term studies [19]. In addition, the study was designed to yield follow-up data on spontaneous sphincter regeneration by transurethral urodynamics one to six weeks after induction of incontinence. To do so, standard urethral pressure profilometry (s-UPP), was employed as used in daily clinical practice. In addition, a novel, high-definition urethral pressure profilometry (HD-UPP) technology was included in this study to validate the s-UPP data obtained from the four cohort 2 and the six cohort 3 animals by a innovative sensor prototype using the HD-UPP method on an exploratory level [17, 29–31]. Moreover, in parallel, the spontaneous tissue recovery was investigated in the pigs with sphincter deficiency by preparing urethral tissue samples from cohort 1 animals as early as one week after SUI induction and up to six weeks after follow-up (Fig. 1).

The original purpose of this feasibility study was to corroborate a urinary incontinence model in GM suitable to prepare the basis for future short- and long-term investigations of novel SUI therapies. Such studies may be suitable to test needle-free transurethral injections of active components in the sphincter muscle using an advanced waterjet technology [23] or to investigate novel transurethral diagnostic procedures, such as HD-UPP in animals without or with cell therapies [17, 29–31]. In GM, however, the spontaneous regeneration of the urethral sphincter complex during follow-up of six weeks was fast and efficient. Thus, GM did not prove to be a suitable model for later incontinence therapy studies. This meant that we had to employ another porcine UI model to generate a significant and sufficient urethral muscle deficiency suitable for future therapy studies. Therefore, we extended our experiments with German landrace (GL) gilts to induce UI, explore the extent of sphincter deficiency and its spontaneous regeneration employing the same methods applied for GM [17].

## Methods

### Animal model of urinary incontinence and wound healing

Female Göttingen minipigs (GM; *n* = 20, mean weight approx. 25 kg, 12 months of age) were obtained with excellent health status and reports directly from the breeder (Ellegaard, Dalmose, Denmark). They were housed prior to surgery under specific conditions in the animal facility in cohorts of four to six gilts in separate pens for two weeks. German landrace gilts (GL; *n* = 6, mean weight approx. 30 kg, 3 months of age) were obtained with excellent health status directly from the breeder (Benz GbR, Bingen, Germany) and housed in the animal facility in one pen for two weeks prior to surgery. Before induction of sphincter deficiency, animals were sedated by pre-medication (atropin: 0.05 mg/kg body weight (bw) i.m. and azaperon: 4.0 mg/kg bw i.m.), followed by anesthesia employing midazolam: 1.0 mg/kg bw i.m. and ketamin (14 mg/kg bw i.m.). The localization and muscular strength of the urethral sphincter complex were determined by cystoscopy under visual control measuring the urethral wall pressure using s-UPP (also known as urodynamics) in sedation (Aquarius TT, Laborie, Portsmouth, NH, USA) as described recently [17]. Then, the animals were put in deep anesthesia (propofol: 5 mg/kg bw i.v. under controlled respiration with 0.6—1.6 Vol% isoflurane, complemented if needed by fentanyl: 5 μg/kg bw i.v.). To establish the animal model of urinary incontinence, sphincter deficiency was induced in deep anesthesia. In cohort 1, GM (*n* = 16) were treated by two lateral electrocauteries (Erbe Hybridknife, mode monopolar cut, effect 4, power 100 W max.; ERBE Elektromedizin, Tuebingen, Germany) just proximal of the wall pressure maximum followed by balloon dilatation as described [17]. In cohort 2, GM (*n* = 4), sphincter deficiency was established by two lateral electrocauteries proximal and two additional lateral electrocauteries distal of the urethral wall pressure maximum, followed by balloon dilatation as described above. Cohort 3 GL (*n* = 6) were treated as cohort 1 GM. Of note, maximum wall pressure was located more distally in the urethra. Immediately after induction of sphincter deficiency and thereafter during follow-up, sphincter function was monitored weekly by s-UPP for up to six weeks. The urine status was determined weekly to monitor, e.g., bleeding, protein, or alike (Combur 10 test, Roche, Pentzberg, Germany). In animals of cohorts 2 and 3, a high-definition prototype device with improved local resolution was used (i.e., high-definition urethral pressure profilometry (HD-UPP) in addition to s-UPP monitoring [17, 30]. From cohort 1 GM, four animals each were sacrificed after 1, 3, 5, and 6 weeks of follow-up to prepare urethral tissue samples for histological analyses. GM of cohort 2 and GL of cohort 3 were sacrificed after 4 and 5 weeks of follow-up, respectively, to prepare urethral tissue samples as well (Fig. 1). During husbandry in the animal facility, animals were observed daily, and their health status was monitored by trained personnel. Depending on the breed, animals were given specific food and water ad libitum. The cohort sizes were calculated based on the assumption that induction of sphincter deficiency generated a drop in urethral wall pressure (i.e., sphincter muscle strength measured by urodynamics) of 60% below control levels. Randomization, blinding, or masking were not included in this feasibility study. The animal study was approved by the Institutional Review Board (State of Baden-Württemberg Animal Welfare Authorities) under file number 35/9185.81–2 / CU01-20G, and performed in full compliance with the ARRIVE 2.0. standards and all other relevant regulations.

### Generation of cryosections and histochemistry

After follow-up, gilts were sedated (see above) and sacrificed (pentobarbital: 45 mg/kg bw, i.v., lethal). The urethral sphincter was prepared, cut open dorsally, suspicious tissue samples were embedded in freezing compound (Tissue Freezing Medium, Leica, Richmond, USA), frozen in liquid nitrogen, and stored at −80 °C. Cryosections were generated (20 μm, Leica CM1860 UV, Leitz, Wetzlar) and mounted on super frost slides. To visualize the urethral tissue, histochemistry was performed using AZAN trichrome- and HE-staining protocols [17]. The samples were evaluated and recorded by microscopy (Zeiss Axio Vert.A1, Zeiss, Oberkochen). Overview pictures were generated by ‘Image Composite Editor’ software (Microsoft, Redmond,WA, USA).

### Statistics

Raw data were used for statistical analyses. All statistical analyses were performed with R Statistical Software (v4.2.2) [32], and the nparLD R package (v2.2) [33] was used for calculations of ANOVA-type statistics reported in the figures. Nonparametric analyses were chosen due to small sample sizes and not normal data distributions. *P*-values < 0.05 were determined as significant and marked in the figures by asterisks (*p* < 0.001 ***; *p* < 0.01 **, *p* < 0.05 *). For the evaluation of data of cohort 1, multiple imputation was performed using the amelia R package (v1.8.1) [34]. 100 imputed data sets were created with a linear time effect, lower bound set to zero, and lags and leads defined as the y-variable “area under the curve (AUC)”. To analyze the results with the nparLD R package, averages of the imputed data sets were built. For better comparison, raw data were normalized to the time point “Before” incontinence induction as 100% for each animal.

## Results

### Sphincter regeneration after moderate injury in Göttingen minipigs

**Fig. 2:**
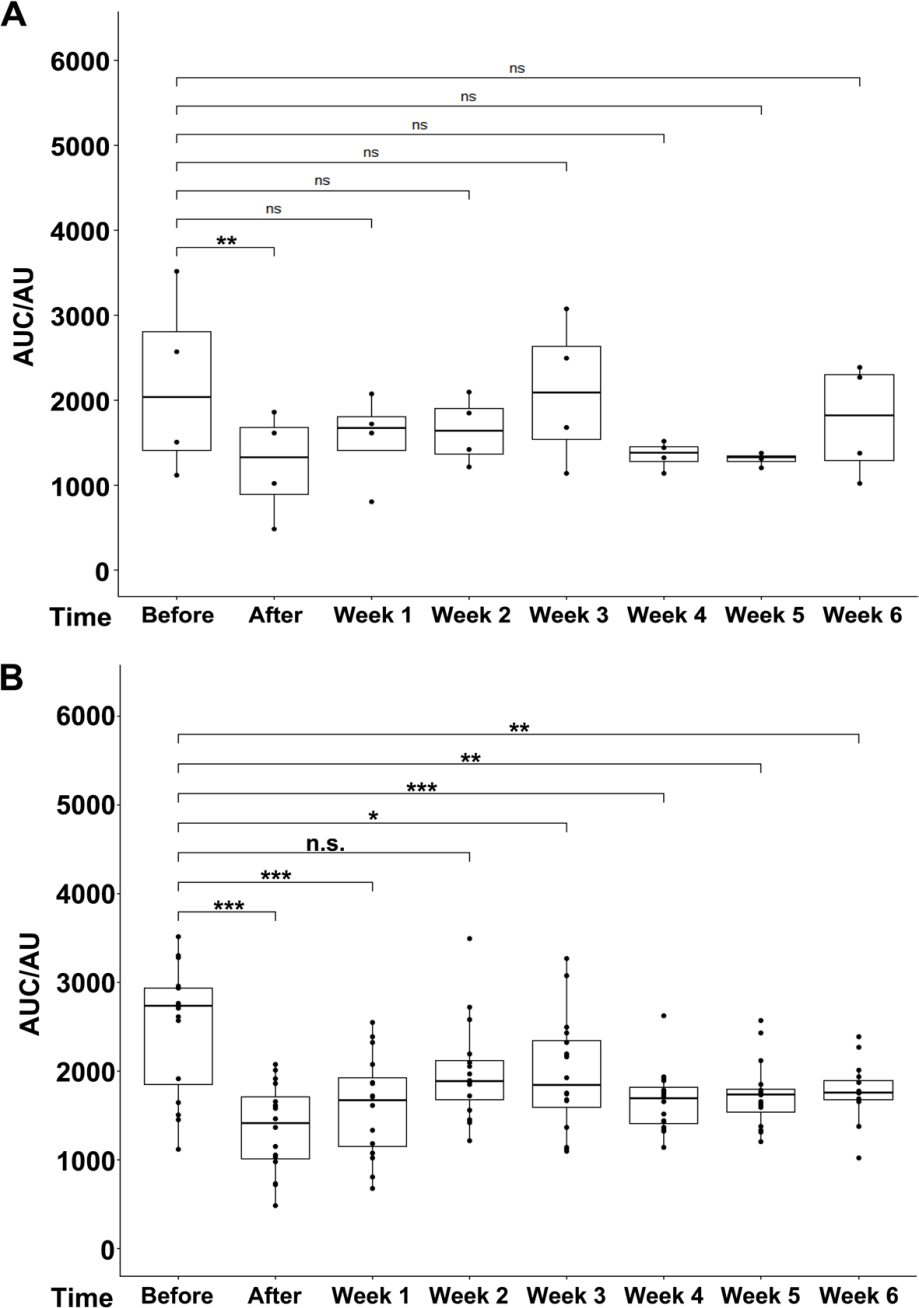
Boxplots of urodynamic measurements of cohort 1 determined with standard urethral pressure profilometry (s-UPP). **A** Boxplots and *p*-values were calculated, and nonparametric analyses are reported for the four GM which completed six weeks of follow-up period. The areas under the curve (AUCs) were obtained from the original measurements. In these analyses, a significant result is computed only between “Before” and “After” surgery (*p* < 0.01; **). All other comparisons were not significant (n.s.). **B** Urodynamic measurements were performed immediately before (Before) and immediately after (After) induction of sphincter deficiency starting with sixteen GM gilts, and weekly during follow-up (week 1–6). The areas under the curve (AUCs) were obtained from the original measurements. Since after 1, 3, and 5 weeks, four GM each were taken out, the missing data were imputed, averages were built, and p-values were calculated from these averages via nonparametric analyses to maintain an artificial/virtual cohort size of sixteen animals throughout the study. Highly significant results (***) were computed for the AUC median pairs “Before” to “After” and “Before” to “Week 1” (*p* < 0.001), respectively. “Before” to “Week 2” was not significant, but “Before” to weeks three to six were significant again, as indicated by asterisks. The x-axes present the time points of urodynamic measurements, the y-axes the urethral wall pressure as AUC in arbitrary units (AU)

Sphincter deficiency was induced in 16 GM of cohort 1 in the area of maximal urethral wall pressure by proximal electrocautery followed by balloon dilatation [17] (Fig. 1). The degree of muscle deficiency was enumerated by s-UPP immediately after induction of sphincter deficiency and during follow-up for a total of six weeks (Fig. 2). When computing the sphincter insufficiency for the four animals completing the study in cohort 1, significant differences were recorded between sphincter function before induction of deficiency and immediately after surgery (Fig. 2A; 60.5% ± 26.6%; *n* = 4; *p* < 0.01). However, as early as one week after surgery and during follow-up, significant differences were not recorded anymore (Fig. 2A). Nevertheless, sixteen gilts entered the study in cohort 1. One, three, and five weeks after surgery four animals each were taken out of the study for histology (Fig. 1). We, therefore, imputed the missing data to maintain a virtual cohort size of 16 GM. In the imputed total population, a significant loss of sphincter function was recorded immediately after induction of incontinence (56.9% ± 19.7%; *n* = 16; *p* < 0.001) and after one week of follow-up (68.8% ± 26.8%; *n* = 16; *p* < 0.001) when compared to the same animal prior surgery (Fig. 2B). Still, no significant difference was calculated for two weeks of follow-up (Fig. 2B; 93% ± 34.6%). Virtually significant differences were computed between the urethral wall pressure before induction of incontinence compared to three to six weeks after surgery (Fig. 2B). Urinary infection, white and red blood cell counts, as well as urine chemistry (pH, glucose, etc.) were monitored throughout the study, and pathological abnormalities were not recorded in 14/16 animals of cohort 1. However, 2/16 animals suffered from urethral infection after induction of sphincter deficiency.

**Fig. 3:**
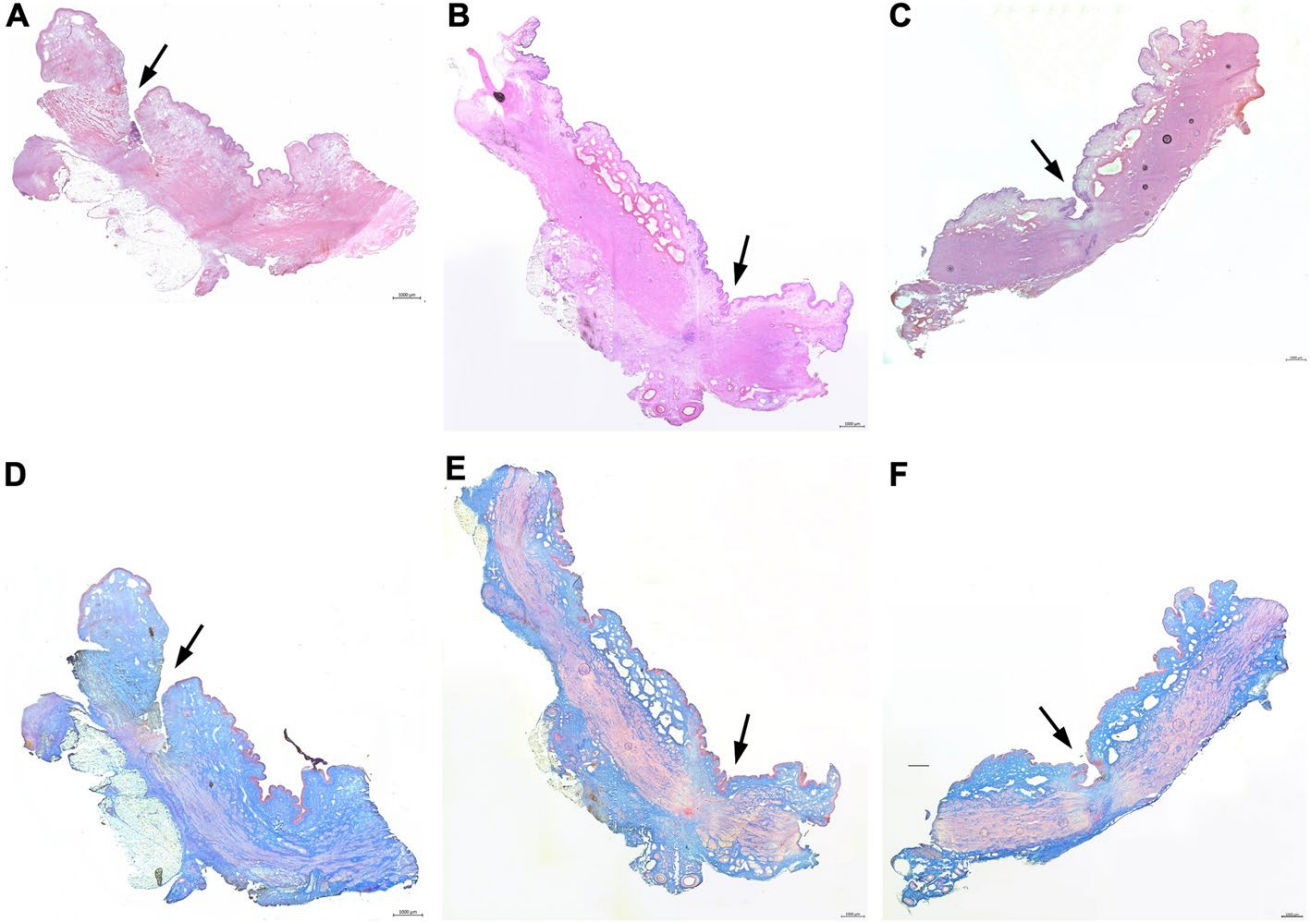
Histological analyses of representative animals of cohort 1 at weeks 1, 3, and 5 after induction of sphincter insufficiency. HE (A-C) and Azan (D-F) staining of consecutive 20 µm-cryosections of one representative animal per time point of cohort 1. **A**,**D**: One week after surgery, histochemistry shows serious injury of the urothelial layer, incision in the mucosa (arrow), and interrupted muscle structures. Mucosal blebs were not found. **B**, **E**: Three weeks after surgery, incisions are not observed anymore, the urethral layer is intact, in the mucosa blebs are found, but the sphincter muscle shows structural irregularities (arrow). **C**, **F**: Five weeks after surgery, the urethral layer is intact, in the mucosa blebs are visible, and the sphincter muscle layer is slightly interrupted in one animal (arrow). Scale bars indicate 1000 µm

From cohort 1, four animals each were sacrificed one, three, and five weeks after induction of incontinence to investigate the sphincter tissue by histology (Fig. 1, 3). Tissue injury was noted one week after electrocautery and balloon dilatation (Fig. 3A, 3D). In the mucosa no blebs were visible. Signs of infiltration of mononuclear cells were not observed (Fig. 3A). After three weeks of follow-up, tissue regeneration was visible by histochemistry in cohort 1 animals. Blebbing of the mucosa was observed (Fig. 3B, 3E). Structural changes and muscular abnormalities in the rhabdosphincter were evident in three of four animals investigated in detail (Fig. 3E). One of the four animals showed no muscular irregularities (data not shown). After five weeks of follow-up, the muscular layer and the mucosa appeared almost normal in cohort 1 animals (data not shown), except for two animals in which each one suggested electrocautery location with still interrupted muscular layer was recorded (Fig. 3C, 3D). These histological analyses of tissue samples (Fig. 3) corroborated the results of the urodynamics indicating a rapid functional sphincter regeneration in GM (Fig. 2B).

### Sphincter regeneration after enhanced injury in Göttingen minipigs

**Fig. 4:**
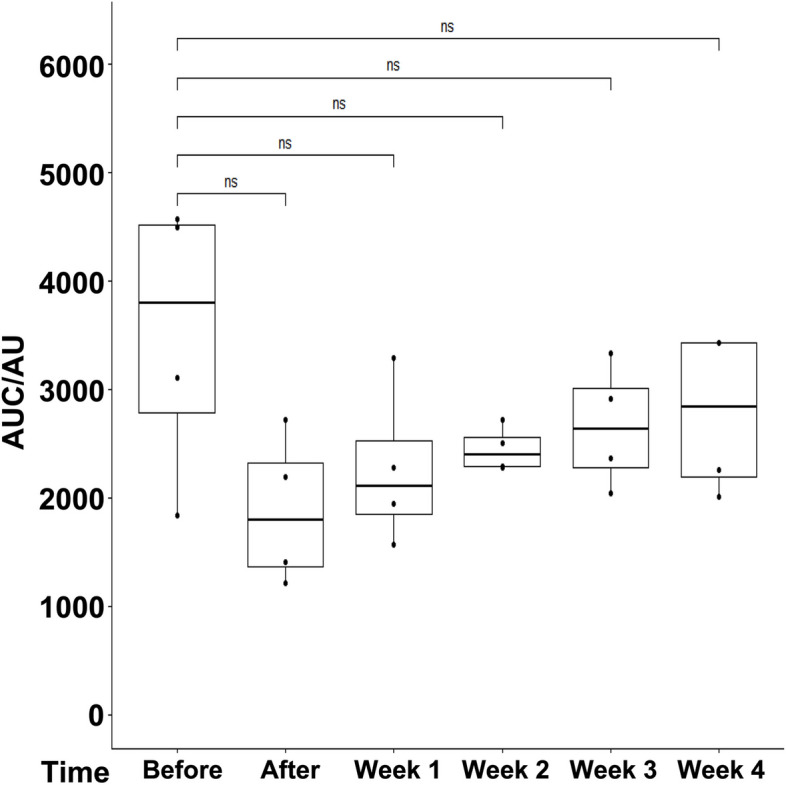
Boxplots of urodynamic measurements of cohort 2 determined with standard urethral pressure profilometry (s-UPP). Urodynamic measurements were performed with standard urethral pressure profilometry (s-UPP) with four cohort 2 animals as described in Fig. 2B. Areas under the curve (AUCs) were calculated from the original measurements. No significant *p*-values were obtained for any of the comparisons. The x-axis presents the time points of urodynamic measurements, the y-axis the urethral wall pressure as AUC in arbitrary units (AU)

**Fig. 5:**
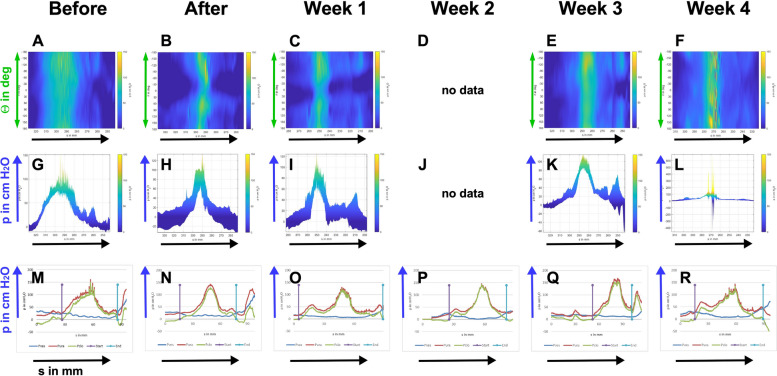
Urethral wall pressure analyses of cohort 2 monitored over four weeks after incontinence induction with two distinct devices. **A – L** Urodynamic measurements were performed with a high-definition urethral pressure profilometry (HD-UPP) prototype immediately before incontinence induction (“Before”), immediately after incontinence induction (“After”), and weekly during follow-up (Week 1–4). One representative animal is depicted. In week 2, no data were measured with the HD-UPP due to technical failure of the prototype. The results of HD-UPP measurements are displayed in two versions. Mode 1 (A-F) displays the urethral wall pressure as three-dimensional graph (stretch “s” in mm on the x-axis, angular position θ = clockwise angle in degrees on the y-axis, and urethral wall pressure “p” in cm H_2_O as a color range from blue (= 0 cm H_2_O) to yellow (= 150 cm H_2_O). Mode 2 (G-L) displays a two-dimensional view of the data produced by HD-UPP. On the x-axis, the stretch in mm is shown, while on the y-axis the urethral wall pressure is displayed in cm H_2_O. Note the different dimensions on the y-axis of the graphs. The color code in modes 1 and 2 did not show any difference over time in the height of urethral wall pressure but indicate a smaller width of maximal wall pressure for “After” and “Week 1", respectively. **M—R:** In addition, urodynamic measurements were performed in all animals by standard urethral pressure profilometry (s-UPP). The graphs show data of the same animal and time points as in A – L. The colored curves represent the vesical pressure Pves (blue) and urethral pressure Pura (red) measured with sensors, and the calculated urethral closure pressure Pclo (green). This data set was used to compute the AUC for Fig. 4. The upright violet start- and the turquoise end- lines indicate the range selected for AUC calculations. The graphs supported the results of A-L

In contrast to our recent study employing GL [17], a functional sphincter recovery in cohort 1 GM was observed as early as one to two weeks after surgery (Fig. 2). We hypothesized that two proximal electrocauteries were not sufficient to establish a robust SUI model in GM. In a small proof-of-principle study, four GM were treated by four electrocauteries, placing two injuries just distal and two just proximal outside the zone of maximal urethral wall pressure, and complementing the electrocauteries by balloon dilatation (Fig. 1). Significant differences in the urethral wall pressure were not recorded after enhanced induction of sphincter deficiency compared to the wall pressure before surgery (Fig. 4). To confirm these unexpected results obtained by s-UPP, urethral wall pressure was also measured by a prototype sensor and the novel HD-UPP technology (Fig. 5). The HD-UPP prototype recorded the urethral wall pressure by eight sensors positioned on the catheter in a spiral-shaped distribution [29–31]. The HD-UPP facilitated a high-resolution measurement as a function of the urethral stretch s (in mm) from the bladder neck and the angular position Θ (in deg) of an individual measuring point. Pressure levels are indicated by colors in two-dimensional (2D) blots (Fig. 5A - F). The green zone of elevated wall pressure with a width of approximately 30 mm indicated the wall pressure maximum and did not disappear nor change color after surgery. However, a narrowing of the (“green”) zone of maximal urethral wall pressure was noted immediately after surgery and during follow-up. The HD-UPP prototype also allows to present the urethral pressure profiles as histograms using the same colors as in the 2D blots (Fig. 5G – L). A difference in the maximal urethral wall pressure was not recorded in animals before surgery and during follow-ups up to four weeks, and pressure levels of approximately 100 cm H_2_O were measured (Fig. 5G – L). The data generated by s-UPP to compute mean differences and statistics (Fig. 4) were utilized to generate pressure histograms as well. The s-UPP histograms confirmed the narrowing of the zone of maximal urethral wall pressure after surgery and during follow-up compared to animals before treatments (Figs. 5G – L). Utilizing s-UPP, slightly higher pressure levels of approx. 150 mm H_2_O were determined (Fig. 5M – R) when compared to HD-UPP histograms (Fig. 5G – L). Both sensor types failed to determine significant differences between animals before versus after induction of sphincter deficiency. Therefore, this proof-of-principle study was terminated after four weeks to comply with animal welfare regulations. As a prototype sensor was employed for HD-UPP in these experiments, differences observed between HD-UPP and s-UPP can be explained by a different calibration of the device. Urinary infection, white and red blood cell counts, as well as urine chemistry (pH, glucose, etc.) were monitored throughout the study, and pathological abnormalities were not recorded.

**Fig. 6:**
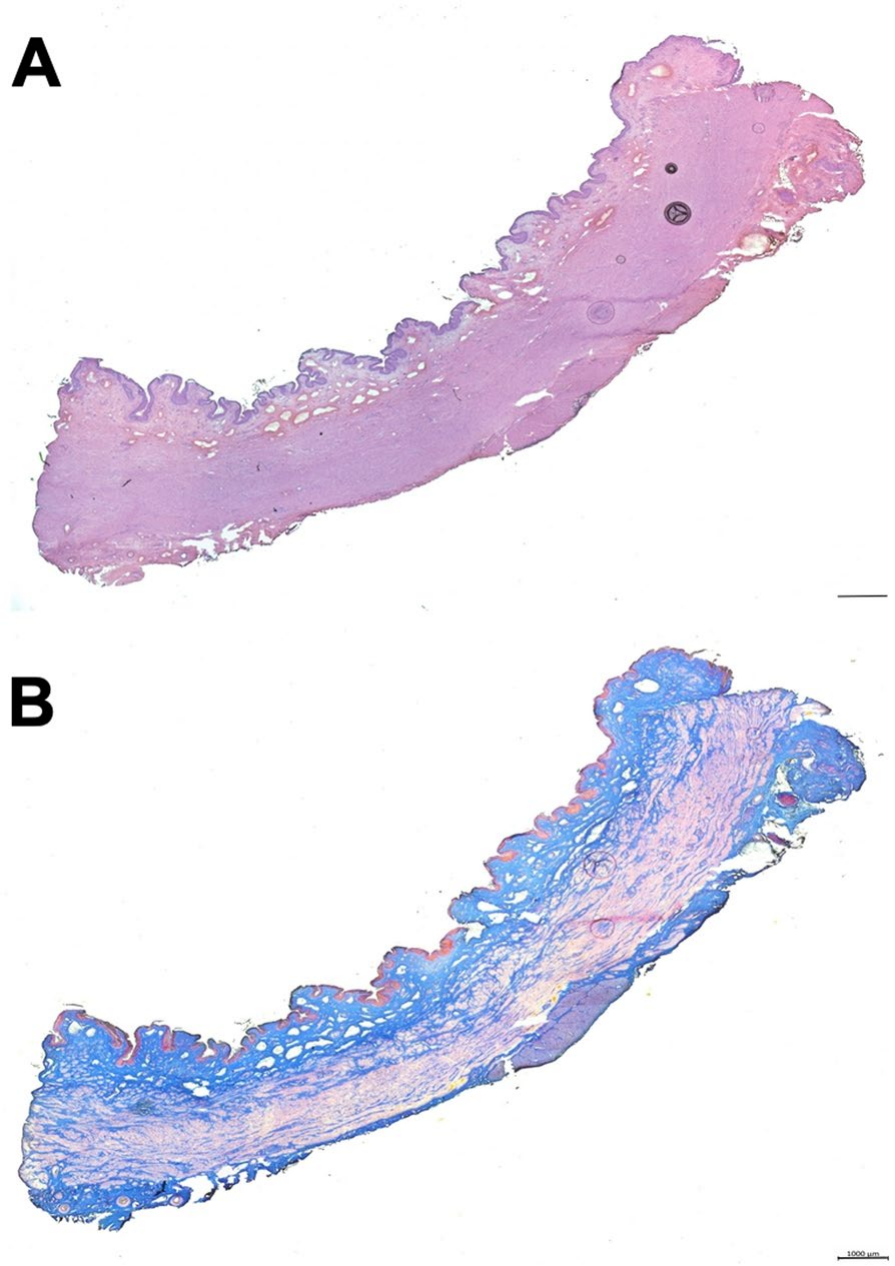
Histological analysis of representative animal of cohort 2. Representative HE (**A**) and Azan (**B**) staining of consecutive 20 µm-cryosections of one representative animal of cohort 2 after four weeks of follow-up. Staining provided no evidence for interrupted muscle structures and no interruption of the urothelial structure resulting from the electrocautery was observed. Small blebs in the mucosa were visible. Scale bars indicate 1000 µm

Histochemistry of sphincter samples from cohort 2 animals of the proximal electrocauteries was performed after the animals’ sacrifice after four weeks of follow-up corresponding to cohort 1 animals. Infiltration of mononuclear cells was not observed. At that time point, apparent signs of tissue injury were not recorded in the urothelial, mucosal, and submucosal layers of the urethra. The sphincter muscle stained with regular patterns (Fig. 6). The results corroborated that GM recovered from induction of sphincter deficiency by balloon dilatation plus fourfold electrocautery quite fast (Figs. 5, 6). Considering GM as SUI model, reliable differences in sphincter recovery were not observed after moderate versus enhanced electrocautery and balloon dilatation (Figs. 2 , 3, 4, 5 and 6).

### Sphincter regeneration after moderate injury in German landrace gilts

**Fig. 7:**
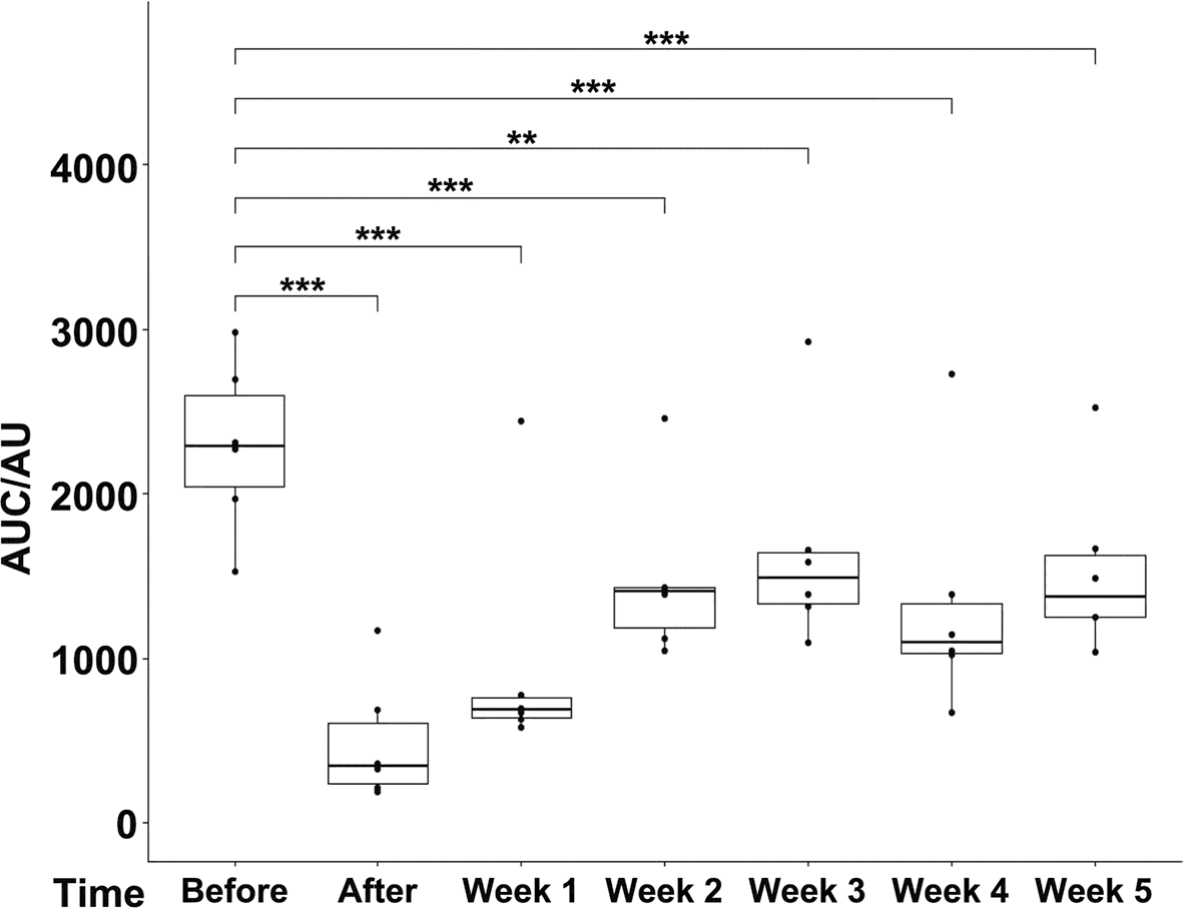
Boxplots of urodynamic measurements of cohort 3 determined with standard urethral pressure profilometry (s-UPP). Urodynamic measurements were performed with standard urethral pressure profilometry (s-UPP) immediately before incontinence induction (“Before”), immediately after incontinence induction (“After”), and weekly during follow-up (Week 1–5). The areas under the curve (AUC) indicating urethral sphincter function were calculated from the original measurements. Significant and highly significant p-values were computed for all comparisons as indicated (**, ***) using “Before” as control. The x-axis presents the time points of urodynamic measurements, the y-axis the urethral wall pressure as AUC in arbitrary units (AU)

**Fig. 8:**
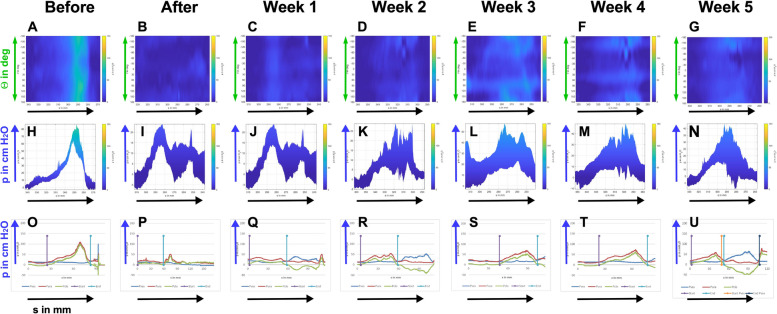
Urethral wall pressures of cohort 3 monitored over five weeks after incontinence induction with two distinct devices. **A** – **N**: Measurements of urethral wall pressure were performed with a high-definition urethral pressure profilometry (HD-UPP) in cohort 3 pigs as described in Fig. 5. One representative animal is shown. Urodynamics with HD-UPP in mode 1 (A-G) display the urethral wall pressures as three-dimensional graphs and in mode 2 (H-N) as two-dimensional histograms. The color code (A – G) and histograms (**H**—**N**) depicted normal sphincter function only before (A, H) but not after surgical treatment nor during follow-up (B – G, I – N). **O**-**U**: In addition, urodynamic measurements were performed in all animals by standard urethral pressure profilometry (s-UPP) as well. The graphs show data of the same animal as in A – N. Details are explained in the legend to Fig. 5. The graphs also support the results of A-N

To verify the improved sphincter regeneration in GM included in this study, sphincter insufficiency was induced in six GL applying the moderate procedure comparable to cohort 1 animals distally in the zone of maximal urethral wall pressure. The animals were observed for five weeks of follow-up (Fig. 1). In contrast to GM, a significant and lasting sphincter muscle deficiency was observed in GL after moderate injury using a double electrocautery plus balloon dilatation. This was evident immediately after surgery and during follow-up (Fig. 7). The mean urethral wall pressure dropped immediately after surgery to 20.8% ± 12.2% (*p* < 0.001). Spontaneous tissue regeneration and wound healing during follow-up elevated the wall pressure to 71.7% ± 19.4% three weeks after surgery. Nevertheless, it remained significantly below levels determined in untreated animals throughout the follow-up of five weeks (*p* < 0.01 to *p* < 0.001; Fig. 7). To corroborate the results obtained by s-UPP after moderate induction of sphincter deficiency, HD-UPP was performed in cohort 3 animals as well (Fig. 8). As explained above, green and yellow colors indicate elevated urethral wall pressure in 2D blots (Fig. 8A – G). Immediately after induction of sphincter deficiency and during follow-up, sphincter muscle activity or urethral wall pressure remained at (“blue”) base levels (Fig. 8B – G). The histogram mode of the HD-UPP determined about 80 cm H_2_0 urethral wall pressure prior to surgery (Fig. 8H), a reduction to about 20 cm H_2_0 (Fig. 8I – K) immediately after surgery and during early follow-up, reaching a peak three weeks after surgery (50 cm H_2_O; Fig. 8L), and leveling off at about 40 cm H_2_O, after four and five weeks, respectively (Fig. 8M, N). The data generated by s-UPP to compute mean differences and statistics (Fig. 7) were utilized to generate pressure histograms as well (Fig. 8O -U). By s-UPP, a urethral wall pressure maximum of 100 cm H_2_O was recorded in animals prior to induction of sphincter deficiency (Fig. 8O). After surgery, the wall pressure dropped below 50 cm H_2_O and remained below this level during follow-up (Fig. 8P – U). Urinary infection, white and red blood cell counts, as well as urine chemistry (pH, glucose, etc.) were monitored throughout the study, and pathological abnormalities were not recorded.

**Fig. 9:**
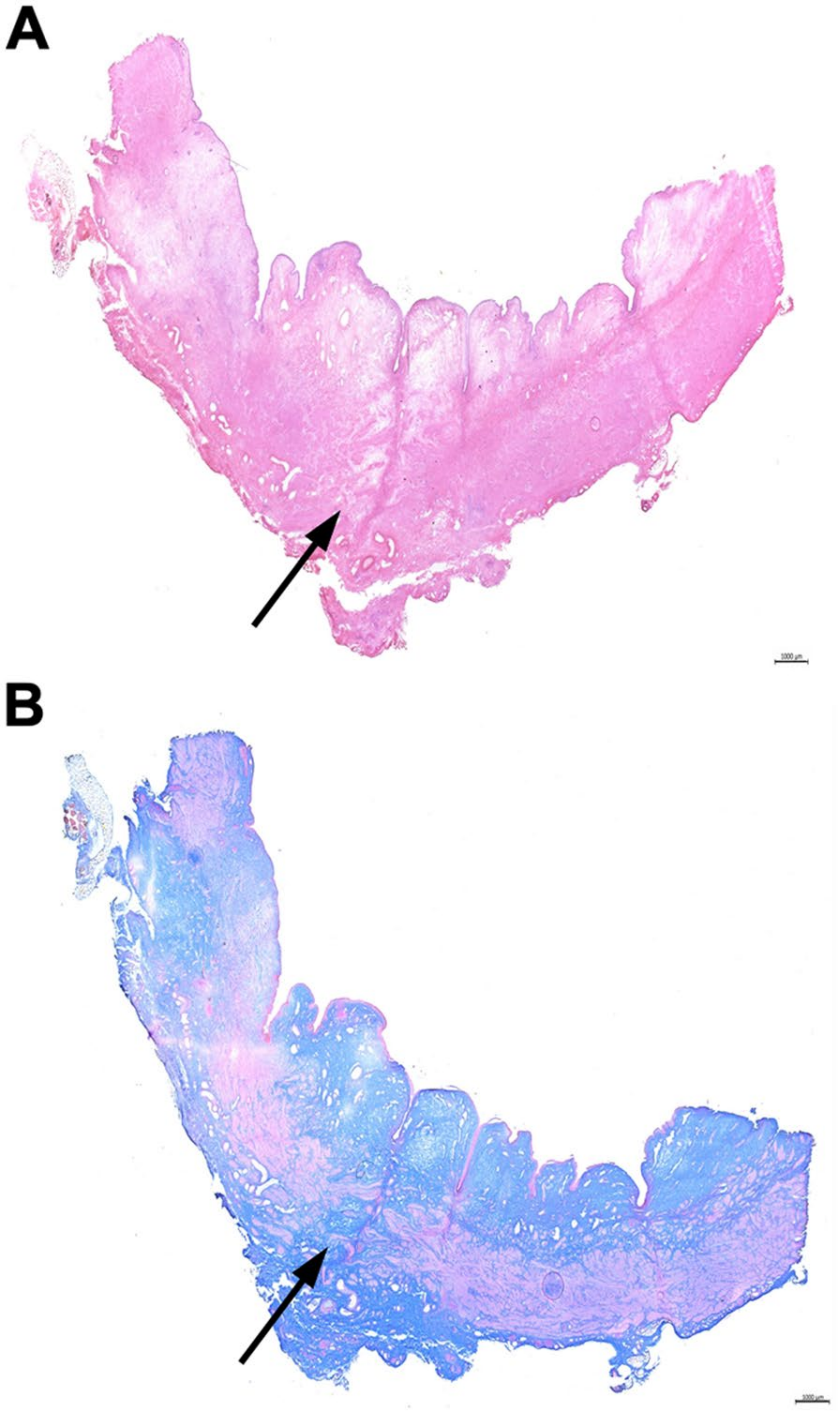
Histological analysis of sentative animal of cohort 3. Representative HE (**A**) and Azan (**B**) staining of consecutive 20 µm-cryosections of one animal of cohort 3 five weeks after induction of sphincter deficiency. Interruption of muscular structures and connective tissue in the zone of the urethral rhabdosphincter muscle were seen by HE and AZAN staining (arrows). Alterations in the mucosa or urothelial layer were not noted. Scale bars indicate 1000 µm

After follow-up and animals’ sacrifice, cryosections of urethral sphincter samples were analyzed by histochemistry (Fig. 9). Infiltration of mononuclear cells was not observed by HE staining, but the tissue integrity presented with irregularities (Fig. 9A). By AZAN staining disruption of the muscular layer and replacement by connective tissue was recorded. Moreover, in the mucosa, no blebs were found suggesting compression of the tissue by balloon dilatation. (Fig. 9A and B). These findings are in line with the results obtained by s-UPP and HD-UPP (Figs. 7, 8). Moreover, it confirmed that moderate induction of sphincter deficiency by double electrocautery followed by balloon dilatation persisted in GL significantly during a follow-up of five weeks. Thus, GL have proven a robust large animal model of stress urinary incontinence.

## Discussion

Porcine models of diseases gained interest recently upon completion of the sequencing of the porcine genome, generation of mutant lines by recombinant technologies, and based on the fact that e.g., size of organs, omnivore metabolism, genetics, and other characteristics are more closely related to humans when compared, for instance, to rodents [35, 36]. Among different porcine breeds, smaller ones such as GM are preferred through their limited growth and weight even after extended follow-up [37]. We, therefore, hypothesized that GM may provide a large animal model of UI to facilitate preclinical studies on the efficacies of different SUI therapies including cell therapies with an extended follow-up but without biasing anatomic changes due to the growth of the animals [19]. However, in contrast to recent studies employing landrace gilts [17, 38], significant sphincter deficiency was recorded in GM only up to one to two weeks after induction of urethral muscle injury. Even enhanced electrocautery followed by balloon dilatation failed to induce a significant sphincter deficiency in GM. In contrast, moderate surgical intervention employing only two lateral electrocauteries of the urethral sphincter yielded a significant, robust, and lasting sphincter deficiency in GL. Moreover, the level of reduction of urethral wall pressure immediately after electrocautery and dilatation was only about 40 – 45% in cohort 1 and cohort 2 gilts when compared to about 80% in cohort 3 animals. This difference could indicate that the tissue elasticity of the sphincter complex, the mechanical resistance to dilatation, vascularization, and other specificities differ in GM in relevant ways from GL. Such characteristics of the breeds may at least in part contribute to the significant difference in SUI induction observed between cohort 1 and 2 animals when compared to cohort 3 animals. Therefore, we suggest that differences in the outcome of this preclinical study were most likely associated with the breed of the animals, including the anatomy of the sphincter.

Differences in the regeneration of the critical contractile compound in our model, the muscle tissues of the urethral sphincter, must be considered when discussing the differences in sphincter regeneration measured. Growth patterns of smooth muscle cells isolated from GM compared to GL provided evidence that cells of GL proliferated faster and seeded scaffolds better than those isolated from GM [39]. Moreover, a higher content of smooth muscle cells was reported in GL arteries compared to GM vessels [40]. In one model for muscular ventricular septal defect therapy, GL presented with good results [41], while other authors claimed Yucatan minipigs as a preferable model for the same therapy [42]. In a surgical model for cardiomyoplasty, GM and GL performed comparably [43]. However, GM granted a superior model for studies of post-myocardial infarction heart failure [44]. This finding would align with our results: better muscle regeneration of GM compared to landrace breeds. In previous studies, left ventricular ejection after myocardial infarction was slightly higher in GM (i.e., 11.3%) when compared to Yorkshire swine [45]. Again, these findings corroborate our study as GM regenerated the sphincter complex faster than GL. Moreover, significant differences between cartilage and chondrocytes were observed between hybrid pigs and GL, respectively [46]. Nevertheless, GM were characteristic of limited repair capacity of osteochondral defects [47]. This indicates that GM do not inherit better tissue regeneration capacities in general compared to other breeds. These studies support the hypothesis that the differences in muscle regeneration in GM and GL explain at least in part the significant differences observed in spontaneous sphincter regeneration.

Another aspect merits discussion as well. Our recent study provided evidence that in GL the maximal urethral wall pressure is localized approximately in the middle of the urethra, about 5 cm distal of the bladder [17]. In GM, the urethral wall pressure maximum was localized somewhat more proximal. Additionally, the two breeds display different urodynamics profiles in untreated conditions as reported recently and confirmed by our results [48]. While GL display a smaller peak in maximal closure pressure, GM represents more with a wider plateau of maximal closure pressure. As electrocautery is a rather pointed injury, it may not harm the wider plateau of the maximal sphincter strength in GM efficiently which might result in a higher loss of wall pressure obtained in GL compared to GM. Therefore, this difference in sphincter injury could contribute to the different outcomes of our incontinence induction and seems essential for the SUI model. One should note that in the four cohort 2 gilts as well as in the six cohort 3 animals, sphincter injury was determined by two distinct methods, by s-UPP and by HD-UPP. Thus, sphincter deficiency as well as its regeneration were measured twice in each animal and time point during follow-up employing two different methods. This procedure corroborated the outcome and enhanced the statistical robustness of the study.

In this study, the sphincter injury by electrocautery targeted the zone of the urethral wall pressure maximum. Here the urethral sphincter complex is predominated by smooth muscle tissue in GL as well as in GM, while striated muscle tissue is found only in the very distal parts of the porcine urethra [17, 49]. The composition of the contractile tissue of the urethral sphincter seems not to discriminate between the two breeds included. The age of the animals could also contribute to the differences observed. However, at least in the human situation, an elevated risk for extended urinary incontinence after mechanical stress by pregnancy or lower pelvic floor muscle trauma upon vaginal delivery is known to correlate with the increased age of the mother [50, 51]. Therefore, the older female GM were expected to suffer from more severe sphincter deficiency when compared to the younger GL. So, the age of the animals seems not a key factor contributing to the shorter and not significant incontinence in GM. The health status of all animals was monitored throughout the study by expert veterinarians. The urine status, body temperature, animal behavior, or increase of weight during follow-up of all animals included provided no evidence for differences of illness nor malady between the breeds (data not shown).

However, differences in urethral vascularization and blood supply could contribute to improved sphincter regeneration in GM. Improved blood supply could also activate local satellite cells or other progenitor cells to facilitate tissue regeneration [52]. To the best of our knowledge, this has not been investigated in detail in GM versus GL, but in GM hepatic blood flow and perfusion were significantly lower than in landrace hybrids [53]. If vascularization is critical in the context of this study, we would expect that sphincter regeneration in GL should not lag significantly behind the functional recovery observed in GM. Nevertheless, detailed studies on urethral vasculature and blood supply are beyond the focus of this study. The composition of the extracellular matrix of the porcine urethra should not be disregarded when investigating differences in sphincter deficiency and regeneration [54]. In human tissue samples of the elderly, collagen content increases and muscle tissue reduction were reported [55]. The sphincter tissue of older GM could therefore be more resistant to tissue injury generated by electrocautery. This hypothesis is also supported by our finding that fourfold electrocautery applied in cohort 2 animals did not cause a significant sphincter deficiency when compared to the twofold electrocautery used in cohort 1 animals. But again, a detailed investigation of the differences in sphincter histology and regeneration in GM on molecular levels is beyond the focus of this study.Other limitations of this exploratory study include the small number of animals investigated in cohorts 2 and 3. In addition, timelines of histology of sphincter tissue injury and recovery were not performed in these two cohorts. This, however, is mainly due to animal welfare requirements. Thus the differences in mechanisms of sphincter regeneration were not explored on a molecular level and remain to be investigated in future studies. Moreover, blinding and randomization of the surgical procedures, follow-up and data evaluation as well as complementation of the experimental cohorts by mock-treated, non-incontinent gilts was not included in this small feasibility study. The bias of results reported that might derive from the open design of this study is acknowledged. Thus, future studies will include larger cohorts, mock-controls, and will be designed as two-armed, double-blind preclinical animal studies to cope with this challenge.

## Conclusion

Göttingen minipigs inherit an elevated potential for rapid sphincter muscle regeneration and therefore failed to serve as robust animal models of urinary incontinence and incontinence therapies. This enhanced potential for muscle regeneration may influence the outcome of animal studies performed with this breed and should be taken into account when comparing studies performed in different breeds.

## Data Availability

Data and materials will be disclosed to colleagues in academia upon written, justified, and comprehendible request to the corresponding author.

## Abbreviations

AUC: Area under the curve
Bw: Body weight
GL: German landrace gilts
GM: Göttingen minipigs
HD-UPP: High-definition urethral (wall-) pressure profilometry
s-UPP: Standard urethral (wall-) pressure profilometry, alias urodynamics
SUI: Stress urinary incontinence
UI: Urinary incontinence

## Declarations

### Consento for publication

No human materials used.
